# An In Vitro Stereomicroscopic Evaluation of Bioactivity between Neo MTA Plus, Pro Root MTA, BIODENTINE & Glass Ionomer Cement Using Dye Penetration Method

**DOI:** 10.3390/ma14123159

**Published:** 2021-06-08

**Authors:** Mohmed Isaqali Karobari, Syed Nahid Basheer, Fazlur Rahman Sayed, Sufiyan Shaikh, Muhammad Atif Saleem Agwan, Anand Marya, Pietro Messina, Giuseppe Alessandro Scardina

**Affiliations:** 1 Conservative Dentistry Unit, School of Dental Sciences, Universiti Sains Malaysia, Kubang Kerian 16150, Kelantan, Malaysia; 2 Department of Conservative Dentistry & Endodontics, Saveetha Dental College & Hospitals, Saveetha Institute of Medical and Technical Sciences University, Chennai 600077, Tamil Nadu, India; 3 Department of Restorative Dental Sciences, College of Dentistry, Jazan University, Jazan 45142, Saudi Arabia; snbasheer@jazanu.edu.sa; 4 Badr Al Samaa Group of Hospitals, Al Khuwair 112, Oman; fazlurrsd@gmail.com; 5 Happy Mouth Dental Clinic, Mumbai 400018, Maharashtra, India; shaik.sufiyan@yahoo.com; 6 Department of Restorative Dentistry, College of Dentistry in Alrass, Qassim University, Ar Rass 52719, Saudi Arabia; dratifagwan@yahoo.com; 7 Department of Orthodontics, University of Puthisastra, Phnom Penh 12211, Cambodia; amarya@puthisastra.edu.kh; 8 Department of Surgical, Oncological and Stomatological Disciplines, University of Palermo, 90133 Palermo, Italy; pietro.messina01@unipa.it

**Keywords:** root end filling, MTA, BIODENTINE, dye penetration, stereomicroscope

## Abstract

The ideal root end filling material should form a tight seal in the root canal by adhering to the cavity walls. Several materials have been used for root end filling. The present study aims to find out and compare the bioactivity of Neo MTA Plus, Pro Root MTA White, BIODENTINE & glass ionomer cement as root end filling materials using 1% methylene blue as tracer. Materials and methods: 80 extracted human permanent maxillary anterior teeth were used in the study. They were divided into four groups. Specimens were sectioned transversely in the cervical area to separate the crown from the root. The root canal was obturated with gutta percha and zinc oxide eugenol sealers. Thereafter, each sample was resected apically by removing 3 mm of the apex and filled with different materials. Samples were kept in buffering solution at 37 °C until the recommended evaluation periods. The specimens were then suspended in 1% methylene blue for 24 h, prior to the analysis. The teeth were then sectioned, and dye penetration was examined, photographed, and evaluated under a stereomicroscope. Results: Vertical dye penetration showed significant differences across different groups. The minimum dye penetration was seen in Neo MTA plus followed by BIODENTINE, Pro Root MTA and maximum in GIC. There was no significant difference in dye penetration between Neo MTA plus and BIODENTINE both at fifteen days and one-month intervals. Conclusion: The present study suggests Neo MTA plus and BIODENTINE should be the preferred material for root end filling.

## 1. Introduction

Removal of all micro-organisms from the root canal is the main objective of root canal treatment [1]. For successful endodontic treatment it is essential to have a material with a conducive nature. In the past few decades there have been many materials that have been introduced with the aim of achieving a favorable tissue response and bioactivity. Complete obliteration of root canal and creation of fluid tight seal are the main factors for effective endodontic care [2]. Endodontic therapy relies on the removal of contaminated channel content accompanied by a suitable compatible material for root canal filling. [3] Despite the development of new endodontic techniques, more effective materials and instruments, the resolution of periapical pathosis is not achieved in certain cases [4]. An ideal root end filling material should form a tight seal in the root canal by adhering with the cavity walls [5]. Manipulations should be simple, and the material should be radiopaque and stable dimensionally. The presence of moisture should not influence an ideal root end filling content. It should be adhesive to dental tissues, non-toxic, well tolerated, bioactive and promote healing by peri radicular tissue [6].

Several root end filling materials have been used such as gutta-percha, Cavit, Super EBA, glass ionomers, amalgam, composite resins, glass ionomer cement (GIC), zinc-oxide eugenol cements, zinc phosphate cements, and mineral trioxide aggregate (MTA). A commonly used material bio-ceramic aggregate with many clinical applications is a mineral trioxide aggregate (MTA) [7]. Bioactivity is an important property associated with bio-ceramic materials. Recently, new bio-ceramic materials have been introduced as an alternative to MTA viz Neo MTA plus and Biodentin. A bioactive material elicits a biological response at the border of material resulting in the development of a bond between tissue and the material. The interaction between a bioactive material and living tissues, results in the formation of an appetite layer (bio-mineralization) at the material–tissue interface [8]. 

The present study aims to find out and compare the bioactivity of Neo MTA Plus, Pro Root MTA White, BIODENTINE and glass ionomer cement as root end filling materials using 1% methylene blue as tracer.

## 2. Materials and Methods

A total of 80 extracted human permanent maxillary anterior teeth were sourced from a department of oral and maxillofacial surgery, the teeth extracted for a reason other than this study. Only teeth with completely formed root apices and free of defects were considered for the purpose of this study. The teeth containing developmental defects, immature apex, root fracture, significant apical curvature, presence of internal resorption, caries, morphological defects, visible cracks, and teeth with previous restorations or endodontic treatment were excluded from the study. For collection of the samples, after extraction, teeth were cleaned of soft tissue debris by an ultrasonic scaler, and inspected for cracks, lesions, or defects, clinically and radiographically. Disinfection of the teeth was carried out by immersion in 5% sodium hypochlorite for 1 hour and subsequent storage in de-ionized water.

### 2.1. Sampling Method

The entire group of samples (*n* = 80) was divided into four main groups based on the root end filling material. Each group carried 20 samples and division was done using random sampling (Table 1).

**Figure 1:**
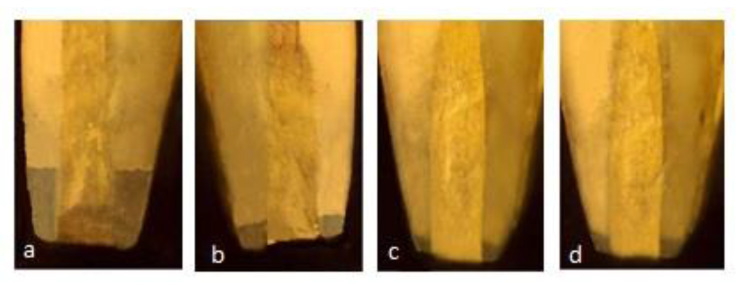
Stereomicroscopic images of leakage in different group at 15 days (**a**) GIC (1.23 mm) (**b**) Proroot MTA (0.45 mm) (**c**) Neo MTA plus (0.08 mm) (**d**) BIODENTINE (0.10 mm).

**Figure 2:**
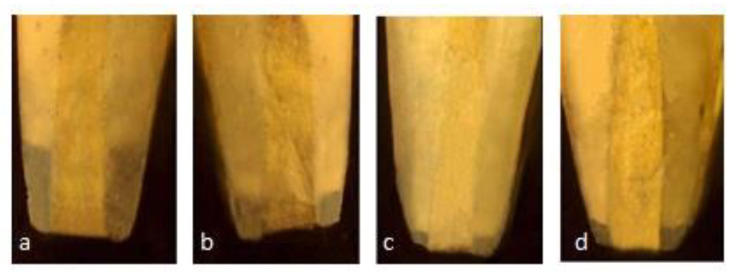
Stereomicroscopic images of Leakage in different group at 1 Month (**a**) GIC (1.35 mm) (**b**) Pro Root MTA (0.53 mm) (**c**) Neo MTA plus (0.08 mm) (**d**) BIODENTINE (0.13 mm).

Each specimen was sectioned transversely in the cervical area to separate the crown from the root, using a diamond disc with a water spray keeping a standard length of the remaining root at 12 mm, to maintain standardization among samples. The working length was established at 1 mm short of the anatomical apex. Biomechanical preparation was done using hand ProTaper and obturation with gutta-percha and zinc oxide eugenol sealer. The coronal access cavity was filled using GIC. This was followed by resection of each sample apically at 90 degrees to the long axis of the root, using a crosscut fissure bur, removing 3 mm of the apex. A 3 mm deep retrograde cavity was prepared using an inverted cone diamond bur, following the manufacturer’s instructions. Samples were kept in the buffering solution at 37 °C for the recommended evaluation period. The specimens were suspended in 1% methylene blue for 24 h prior to the analysis. Following these steps, the samples were rinsed with running water for 15 min. Clear acrylic blocks were made to stabilize the samples. Each specimen was sectioned transversely in the cervical area, maintaining a standard root length of 12 mm for standardization purposes. Then, these specimens were examined under a stereomicroscope using 2× magnification. (Figure 1 and Figure 2).

### 2.2. Evaluation

The dye penetration was independently evaluated by two people not associated with the study, using standardized photographs. The evaluation was carried out based on predetermined criteria. While dye penetration may be an older method of evaluation it is still considered one of the safest as there is no radiation exposure or harmful chemicals involved. 

### 2.3. Statistical Analysis

The collected data was subjected to statistical analysis by using one-way analysis of variance (ANOVA) and intergroup comparison was carried out using Student’s paired *t*-test.

## 3. Results

**Figure 3:**
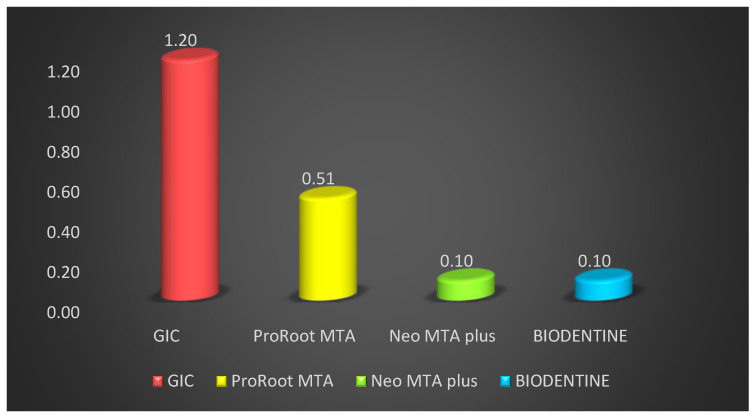
Comparison of the sealing activity of different root end filling material at 15 days.

The teeth were examined for vertical dye penetration in different root end filling materials. Table 1 shows microleakage values for different groups after fifteen days. Comparison of different groups showed that vertical die penetration was seen at the maximum depth in GIC cement, followed by ProRoot MTA, BIODENTINE, and minimum was seen in Neo MTA plus. The mean vertical depth of penetration in GIC cement was maximum at 1.20 ± 0.10 mm followed by ProRoot MTA 0.53 ± 0.16 mm, BIODENTINE 0.10 ± 0.03 mm and minimum in Neo MTA plus is 0.10 ± 0.04 mm. Penetration among root end filling materials showed a statistically highly significant difference in vertical depth of penetration among sealer with F = 248.03 and *p* value = 0.01. Post hoc Tukey test was performed to evaluate the differences in penetration depth between all four groups (Table 2) (Figure 3).

**Figure 4:**
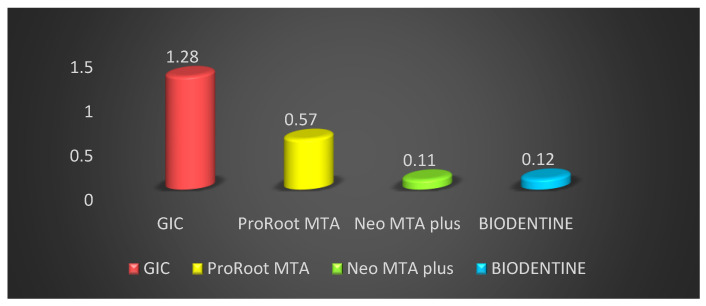
Comparison of the sealing activity of different root end filling materials at one month.

The comparison of microleakage at one month for different groups is shown in Table 2. Also, at one month the comparison of different groups demonstrated that the vertical die penetration was seen at maximum depth in GIC cement, followed by ProRoot MTA, BIODENTINE, and the minimum was seen in Neo MTA plus. The mean vertical depth of penetration was maximum in GIC cement at 1.28 ± 0.08 mm, followed by ProRoot MTA 0.57 ± 0.18 mm, BIODENTINE 0.12 ± 0.04 mm, and the minimum was in Neo MTA plus is 0.11 ± 0.03 mm. Penetration among root end filling materials depicted statistically high significant difference in the vertical depth of penetration among sealers with F = 279.30 and *p* value = 0.01. Post hoc Tukey test was also performed to evaluate the differences between individual groups and indicated a statistically significant difference in penetration depth between all four groups (Table 3). The results revealed that the dye penetration was minimum in Neo MTA plus followed by BIODENTINE, ProRoot MTA and maximum for GIC when used as root end filling material both at fifteen days and one-month intervals (Figure 4).

**Figure 5:**
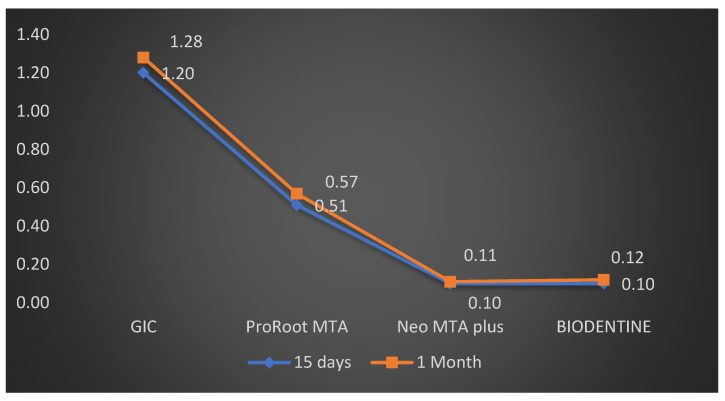
Comparison of sealing ability in all four groups between 15 days and one month.

**Figure 6:**
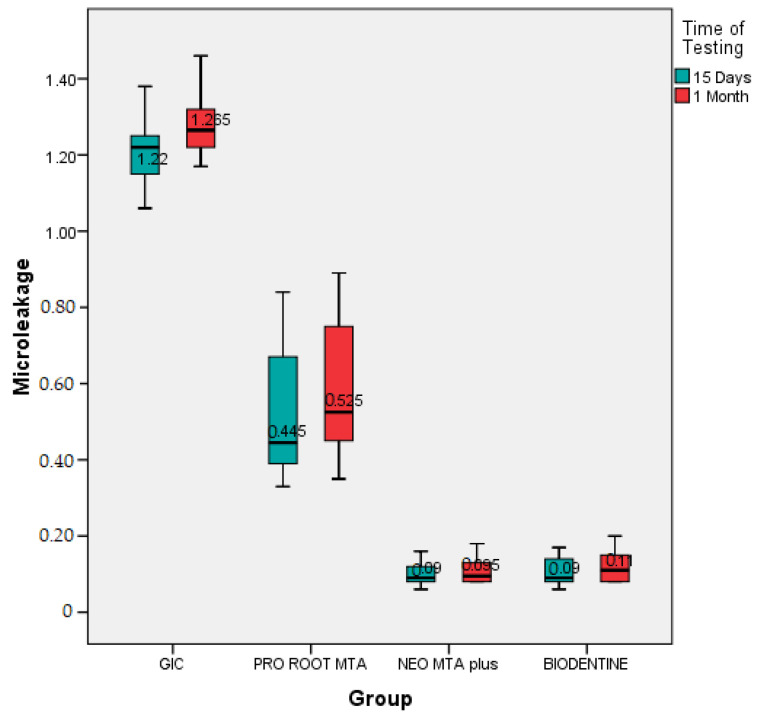
Box-and-whisker plot analysis to compare micro-leakage across the four root end filling materials under consideration.

The intra group comparison between fifteen days and one month was confirmed with the help of paired t-test. Among the GIC group, the dye penetration at one month was 1.28 ± 0.08 mm, which was not significantly greater than fifteen days (1.20 ± 0.10 mm), with *p* value = 0.04 (Table 3). The result for ProRoot MTA showed that the dye penetration at one month was 0.57 ± 0.18 mm but it was not significantly greater than fifteen days (0.53 ± 0.16 mm), with *p* value = 0.67 (Table 3). The intragroup comparison for Neo MTA showed that the dye penetration at one month was 0.11 ± 0.03 mm, which was slightly less compared to fifteen days (0.10 ± 0.03 mm), with *p* value = 0.91. The intragroup comparison for BIODENTINE showed that the dye penetration at one month (0.12 ± 0.04 mm) which was slightly less as compared to seven days (0.10 ± 0.04 mm), with *p* value = 0.89 (Table 4) (Figure 5). A box-and-whisker plot was also designed to evaluate whether the results were skewed, and it was observed that the highest and lowest reported microleakage from the GIC group was higher than that seen in the other groups. Also, the lowest median microleakage was seen in the Neo MTA plus group compared to the others. This confirmed the findings from the other tests that there was significant microleakage in the GIC group over fifteen days as well as one month (Figure 6).

## 4. Discussion

The aim of carrying out surgery in the peri-radicular region is to reach the affected area, to assess the anatomy of the root circumference and the root canal and to put a biocompatible seal in the form of a root end fill, which promotes periodontal regeneration. Many substances have been used as filling materials for the root end. Handling characteristics, biocompatibility, apical seal, and long-term clinical performance will determine the selection of a root end filling material [9]. Glass ionomer cement (GIC) is the most widely used cement in dentistry. When compared with other water-based cements, their strong mechanical and optical characteristics make them superior material. Moreover, the cement releases fluoride and is biocompatible over a longer time, making the GIC a useful dental material. However, GIC has some drawbacks during the early stage of setting such as high brittleness and sensitivity to moisture [3]. The glass ionomer cement forms a weak bond with the tooth [4]. However, Kokub says that a stronger chemical link could be established between GIC and teeth by using acid etching and resin modified glass ionomer cement (RM-GIC) [10].

In the present study, each specimen was sectioned transversely in the cervical area to separate crown from the root, keeping a standard length of the remaining root at 12 mm, to maintain standardization among samples. The most widely used root end filling technique is the dye penetration method, which was used in the present analysis. Because dyes can be easily stored, applied, and have their penetration assessed quantitatively, no reactive chemicals are used along with no radiation; hence, the method is safe, and the technique is highly feasible and easily reproducible [11]. However, the dye penetration method is said to have certain drawbacks include small size of the dye molecules when compared to the bacteria. The various advantages of methylene blue compared to other dyes have influenced researchers to use it as medium. It is cost-effective, easy to manage, and strong in color. This coloring has many drawbacks, for example, dissolution during demineralization and clearing processes; in some cases, the maximum point of penetration is also difficult to observe [12].

In our study, the least dye penetration was observed with Neo MTA plus followed by BIODENTINE Pro Root MTA, and GIC. It denotes that bio-ceramic materials have better sealing ability compared to GIC. The results of the present study show that both Neo MTA plus and BIODENTINE have almost similar figures in the dye penetration after seven days and one month with a statistically insignificant difference for Neo MTA. It could be attributed to similarities in their components, their setting reactions, and bioactive properties. Bio-ceramic materials possess this better sealing ability because of their biocompatible and bioactive nature. In general, the water inherent in the dentinal tubules is used by bio-ceramic sealers to initiate a hydration reaction, minimizing the setting time [13] Neo MTA plus is a tricalcium silicate-based material with tantalum as opacifier. Dammaschke et al. found that Neo MTA plus has sulfur in greater amounts (0.2 ± 0.02), as well as gypsum [14]. Li et al. concluded that gypsum plays an important role in hardening the material by producing hydrous calcium aluminium sulfate(ettringite) during the setting reaction [15].

Kokte and Pawar in 2012 concluded that Biodentin showed the least microleakage in comparison to GIC and MTA, similar to the present study (2). Although the Pro Root MTA seems to be the favorite material of its kind, the cement has many disadvantages. Karabucak et al. stated that compared to other root end filling materials, the handling characteristics of Pro Root MTA are difficult, and that they can eventually cause discrepancies in the procedure [16]. The result of the present study shows that the filling with Neo MTA plus does not produce significant differences in mean microleakage at one week and one month. In their research, Kubo et al. argued that the moisture hydration of the MTA powder would increase compression strength and decrease leakage [17]. The result of Sarkar et al. shows that even in fluid presence MTA has the capability of precipitating hydroxyapatite crystals and may be an important factor in leakage minimization [18].

Various studies show that the minor variation in the lower microleakage values of MTA might be ascribed to its superior marginal sealing ability, resulting from its hydrophilic properties and formations of an interfacial layer between the material and dentin. This layer decreases the chances of marginal percolation and offers encouraging clinical success over the longer term [19]. Bioactive materials have been studied for their biocompatibility and have shown release of varying levels of zirconium, calcium, tungsten and silicone [20]. It has been shown in previous studies that NeoMTA Plus and BIODENTINE demonstrate suitable cytocompatibility with the human dental pulp cells as well as having particularly good cell migration rates [21,22]. In terms of dental bridge formation and maintenance of a vital pulp, both NeoMTA Plus and BIODENTINE have been found to be exceptionally reliable. Most of these materials differ in terms of ease of handling, binding quality, quality of the dentin bridge and protocols, which must be studied extensively to obtain further details. There are various factors responsible for micro-leakage such as: dimensional stability of the material, response to tissue fluids, adaptation and handling and electro-chemical nature. The present study is in vitro in nature and the result cannot be directly applied to the patients. However, the study opens the path for further research to study not only the sealing ability but also other related physical properties, as well as critical manipulative steps.

## 5. Limitations

The dye penetration method is said to have certain drawbacks, including the small size of the dye molecules when compared to bacteria. The various advantages of methylene blue compared to other dyes have influenced researchers to use it as a medium. It is cost-effective, easy to manage, and strong in color. This color strength has many drawbacks, for example dissolution during demineralization and clearing processes; in some cases, the maximum point of penetration is also difficult to observe. Also, the focus of this study was a comparison of the micro-leakage across different materials. It would be good to consider conducting future studies with other physical and chemical properties as variables to develop a better understanding.

## 6. Conclusions

The study concluded that microleakage was present in all the samples. The microleakage was minimum in Neo MTA plus and Biodentin while the maximum microleakage was found in the GIC group. The present study suggests Neo MTA plus and BIODENTINE should be the preferred materials for root end filling.

## Figures and Tables

**Table 1:** Grouping of samples (*n*—total number of samples).

Groups (*n*-20)	After 15 Days (*n*-10)	After 30 Days (*n*-10)
GIC	1a	1b
Pro Root MTA	2a	2b
Neo MTA plus	3a	3b
Biodentin	4a	4b

**Table 2:** Comparison of the sealing activity of different root end filling materials after 15 days (*n*—total number of samples).

Groups	*n*	Mean	Std. Deviation	F Value	*p* Value	Post Hoc Tukey Test
GIC	10	1.20	0.10	248.03	0.01 (HS)	GIC > Pro Root MTA > BIODENTINE ≈ Neo MTA plus
ProRoot MTA	10	0.51	0.16			
Neo MTA plus	10	0.10	0.03			
BIODENTINE	10	0.10	0.04			
Total	40	0.42	0.36			

ANOVA, *p* ≤ 0.05 = significant (s), *p* ≤ 0.01 = Highly significant (HS).

**Table 3:** Post hoc analysis to demonstrate differences between the sealing activity of different root end sealing materials after one month (*n*—total number of samples).

Groups	*n*	Mean	Std. Deviation	F Value	*p* Value	LSD Post Hoc Test
GIC	10	1.28	0.08	-	-	GIC > Pro Root MTA > BIODENTINE ≈ Neo MTA plus
ProRoot Mta	10	0.57	0.18	-	-	
NEO MTA plus	10	0.11	0.03	-	-	
BIODENTINE	10	0.12	0.04	279.30	0.01	

ANOVA, *p* ≤ 0.05 = significant (s), *p* ≤ 0.01 = Highly significant (HS).

**Table 4:** Intergroup comparison of microleakage in GIC group between fifteen days and one month.

Groups	Time Period	Mean	Std. Deviation	t Value	Sig.
GIC	15 Days	1.20	0.13	1.90	0.07
	1 Month	1.28	0.09		
PRO ROOT MTA	15 Days	0.51	0.14	0.87	0.67
	1 Month	0.57	0.18		
NEO MTA plus	15 Days	0.10	0.03	0.06	0.91
	1 Month	0.11	0.02		
BIODENTINE	15 Days	0.10	0.03	0.10	0.89
	1 Month	0.12	0.02		

Paired *t*-test, *p* ≤ 0.05 = significant (s), *p* ≤ 0.01 = Highly significant (HS).

## Data Availability

Any data related to the study can be provided on reasonable request.
